# Generative AI-Assisted Preschool Instruction and Children’s Creative Learning Engagement: The Mediating Role of Autonomous Motivation and the Buffering Role of Perceived Teacher–Child Interaction Quality

**DOI:** 10.3390/bs16081293

**Published:** 2026-07-29

**Authors:** Qinyu Guo, Zhenyu Gao

**Affiliations:** 1 Faculty of Education and Human Development, The Education University of Hong Kong, Hong Kong 999077, China; s1162283@s.eduhk.hk; 2 Jing Hengyi School of Education, Hangzhou Normal University, Hangzhou 311121, China

**Keywords:** generative AI, preschool education, creative learning engagement, autonomous motivation, perceived teacher–child interaction quality, moderated mediation

## Abstract

Drawing on Self-Determination Theory (SDT) and the Classroom Assessment Scoring System (CLASS) framework, this study tested a moderated mediation model in which two dimensions of GenAI-assisted teaching predict children’s creative learning engagement through autonomous motivation. Cross-sectional survey data collected from 486 preschool teachers across 52 kindergartens in three Chinese provinces were analysed with structural equation modelling and bias-corrected bootstrap procedures. AI-enhanced interactive pedagogy was positively associated with autonomous motivation, whereas AI-dependent passive instruction was negatively associated with it; only passive instruction exhibited a direct negative link with creative learning engagement. Autonomous motivation fully mediated the interactive pedagogy pathway and partially mediated the passive instruction pathway. Perceived teacher–child interaction quality significantly moderated the passive instruction to motivation path, attenuating the indirect association to non-significance at high levels of perceived interaction quality. Because all constructs were assessed via teacher self-report at a single time point, the observed associations reflect teachers’ perceptions rather than objectively measured behaviours, and causal inference is not warranted. The study offers theoretical and practical insights for designing developmentally appropriate, teacher-scaffolded GenAI integration in preschool education.

## 1. Introduction

Generative artificial intelligence (GenAI) technologies have entered educational settings at an unprecedented pace since 2022, and early childhood education is no exception. International reviews documented a growing yet nascent body of empirical work, with most studies addressing higher education or K–12 contexts and only a small fraction focusing on the preschool years (Alfarwan, 2025; Chiu, 2024; Kanders et al., 2024; Ljungcrantz, 2026; Nikolopoulou, 2025; Su & Yang, 2022). Because learning experiences during the preschool period have lasting effects on cognitive, creative, and socioemotional development (Hamre et al., 2013; Pianta & Hofkens, 2023), it is essential to understand whether and how GenAI tools enhance or undermine these outcomes.

In the Chinese context, kindergartens navigate a dual imperative: adopting GenAI to enhance interactivity, personalisation, and creative richness while preventing the displacement of hands-on play, peer interaction, and teacher-mediated learning (Chen, 2025; Su & Yang, 2024; Su et al., 2024). This imperative reflects the well-documented developmental distinction between active, interactive technology use and passive screen consumption (Herodotou, 2018; Strouse et al., 2018). Chinese preschool education operates within a centralised accreditation system with specified teacher–child ratios and quality monitoring (Li et al., 2026; Su et al., 2024), contextual features that should be considered when evaluating generalisability.

Two significant gaps remain. Most existing studies treat GenAI-assisted teaching as a unidimensional construct, without distinguishing between interactive applications (e.g., collaborative story generation, AI-scaffolded creative play) and passive applications (e.g., unsupervised screen time with AI-generated content) (Chiu, 2024; Kanders et al., 2024; Ljungcrantz, 2026), potentially obscuring asymmetric associations with children’s motivation and learning (Herodotou, 2018; Strouse et al., 2018). Meanwhile, the motivational pathway identified by Self-Determination Theory (SDT) as the central route through which environmental conditions translate into engagement (Guay, 2022; Ryan & Deci, 2017; Vansteenkiste et al., 2020) has not been tested in the context of GenAI use in preschool settings.

A potentially decisive boundary condition is the quality of teacher–child interaction. The Classroom Assessment Scoring System (CLASS) framework has established that interaction quality is the strongest classroom-level predictor of young children’s academic, social, and self-regulatory outcomes (Hamre et al., 2013; Pianta & Hofkens, 2023). Whether perceived teacher–child interaction quality can buffer the motivational costs of passive GenAI instruction has not been empirically tested. The present study adapts CLASS to a self-report format; the construct more closely approximates teachers’ pedagogical self-perceptions than objectively observed classroom behaviour, a distinction maintained throughout by using the term “perceived interaction quality.”

The present study makes three contributions. Conceptually, it separates AI-enhanced interactive pedagogy from AI-dependent passive instruction, operationalising the active versus passive distinction highlighted in developmental research but not yet applied to GenAI education (Jiang et al., 2025; Liang et al., 2023). Mechanistically, it positions autonomous motivation as the SDT-based mediator linking different GenAI teaching modes to children’s creative learning engagement. Contextually, it introduces perceived teacher–child interaction quality as a moderator of the passive instruction to motivation path. The study relies entirely on teacher self-report; this single-informant design means the observed associations may be influenced by shared rater variance, a limitation addressed in the Discussion. The remainder of the paper develops the theoretical framework and hypotheses (Section 2), describes the methodology (Section 3), reports the results (Section 4), and discusses implications and limitations (Section 5 and Section 6).

## 2. Literature Review and Hypothesis Development

### 2.1. Theoretical Foundation and Framework

The conceptual model is grounded in Self-Determination Theory (SDT) (Ryan & Deci, 2017; Vansteenkiste et al., 2020), which posits that motivation and engagement are shaped by the satisfaction of three basic psychological needs: autonomy, competence, and relatedness. Environments supporting these needs foster autonomous motivation (intrinsic motivation and well-internalised extrinsic motivation), which predicts deeper engagement and creative exploration (Chiu, 2021; Guay, 2022). Environments that thwart need satisfaction push motivation toward controlled or amotivated forms, predicting shallow engagement (Vansteenkiste et al., 2020).

An important consideration is that current GenAI tools are unlikely to serve all three needs equally. Most GenAI applications prioritise content generation and adaptive challenge, supporting autonomy and competence but lacking the emotional attunement required to satisfy relatedness needs (Pianta & Hofkens, 2023; Ryan & Deci, 2017). This asymmetry reinforces the importance of examining teacher–child interaction quality as a boundary condition.

Autonomous motivation is operationalised as a unidimensional composite rather than being disaggregated into intrinsic motivation and identified regulation subtypes (Guay, 2022; Ryan & Deci, 2017), reflecting the less differentiated nature of preschool children’s motivation (Vansteenkiste et al., 2020), the constraints of teacher-report measurement, and the CFA results. This simplification is acknowledged as a limitation.

A complementary framework is the Teaching Through Interactions (TTI) model underlying CLASS (Hamre et al., 2013; Pianta & Hofkens, 2023). It identifies emotional support, classroom organisation, and instructional support as primary mechanisms through which classroom environments influence developmental outcomes. The association of any instructional technology with children’s engagement is filtered through the interaction ecology the teacher creates. The present study adapts CLASS to a self-report format; the construct is best understood as teachers’ perceptions, which are conceptually related to but empirically distinct from observational CLASS scores.

**Figure 1:**
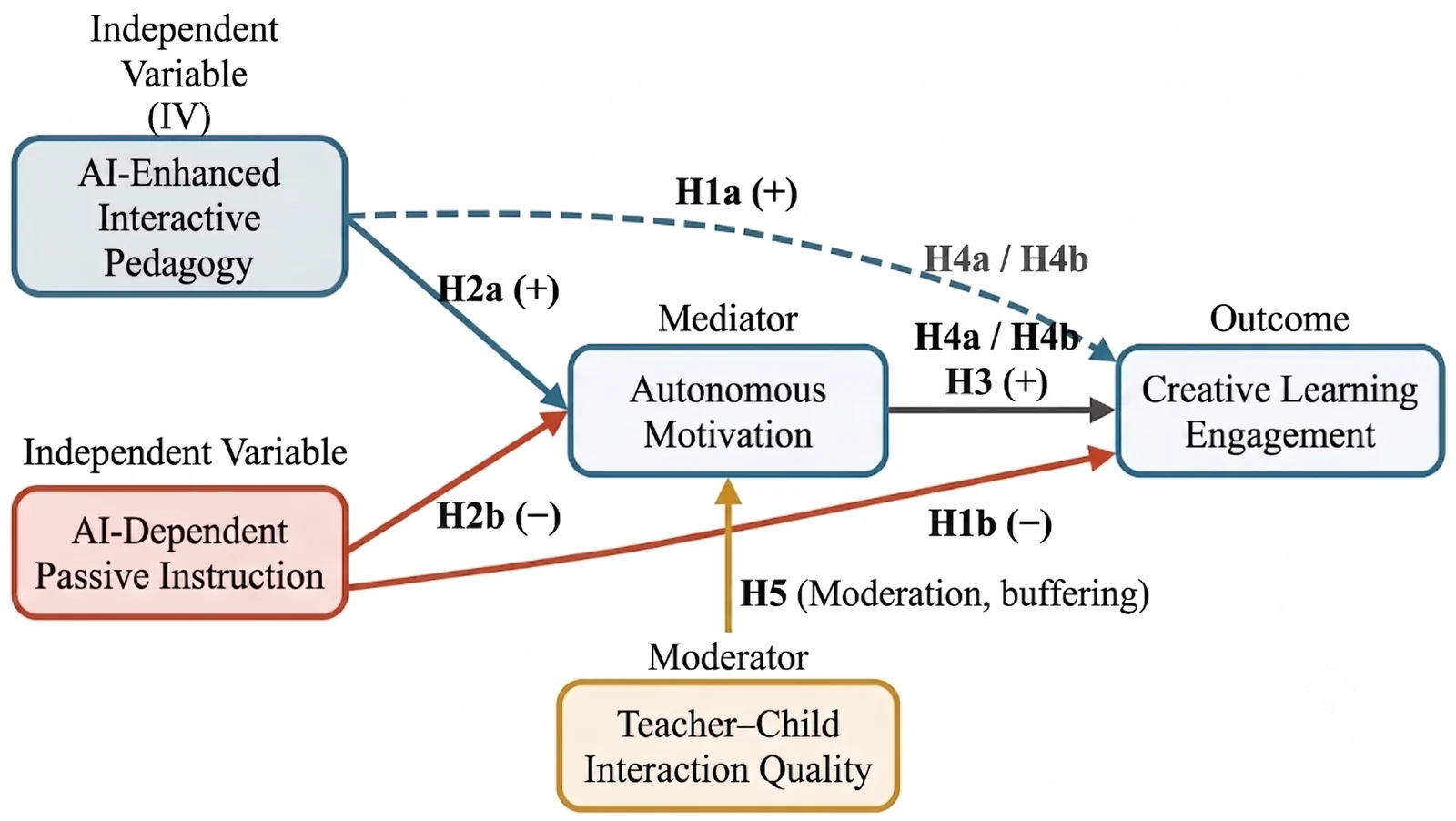
Conceptual framework of the moderated mediation model.

Bringing these strands together yields the conceptual model in Figure 1. AI-enhanced interactive pedagogy captures the extent to which teachers use GenAI tools as co-creative, scaffolding partners (e.g., co-creating stories, facilitating child–AI–teacher collaborative activities) (Nikolopoulou, 2025; Su & Yang, 2024; Su et al., 2024). AI-dependent passive instruction captures the extent to which teachers rely on GenAI in ways that displace direct interaction and increase passive screen consumption (e.g., allowing children to watch AI-generated content without guided discussion) (Herodotou, 2018; Kanders et al., 2024; Strouse et al., 2018). Autonomous motivation is the SDT-based mediator. Perceived teacher–child interaction quality (self-report CLASS adaptation) is the moderator of the passive instruction to motivation pathway.

Perceived teacher–child interaction quality is hypothesised to buffer the path from AI-dependent passive instruction to autonomous motivation.

### 2.2. GenAI-Assisted Teaching and Creative Learning Engagement

Creative learning engagement refers to the behavioural, cognitive, and affective involvement of young children in learning activities requiring imagination and flexible thinking (Amabile & Pillemer, 2012; Fredricks et al., 2004), manifested through sustained attention during open-ended activities, generation of novel ideas, willingness to experiment, and positive emotional responses to creative challenges (Berson & Berson, 2024; Chen, 2025).

Active, interactive uses of digital tools are associated with deeper engagement and stronger motivation, whereas passive, consumption-oriented uses are associated with shallower attention and lower intrinsic interest (Herodotou, 2018; Nikolopoulou, 2025; Strouse et al., 2018). In the GenAI literature, interactive applications have been linked to increased curiosity and engagement, whereas over-reliance on AI-generated content raises concerns about passivity (Chiu, 2024; Guo et al., 2025; Jiang et al., 2025). In the preschool context, AI-generated interactive stories enhanced engagement when embedded in teacher-guided activities, but unsupervised AI use risked displacing relational processes central to early learning (Chen, 2025; Su et al., 2024). We therefore propose:

**H1a.**  *AI-enhanced interactive pedagogy is positively associated with children’s creative learning engagement.*

**H1b.**  *AI-dependent passive instruction is negatively associated with children’s creative learning engagement.*

### 2.3. Autonomous Motivation as a Mediator

Autonomous motivation represents the extent to which an individual engages in an activity out of genuine interest or personally valued goals (Guay, 2022; Ryan & Deci, 2017), and is a key predictor of sustained attention, exploratory behaviour, and creative problem-solving in early childhood education (Chiu, 2021; Vansteenkiste et al., 2020).

Interactive AI pedagogy, which involves children in dialogic co-creation and imaginative exploration, is theorised to support autonomy and competence through choice, scaffolded challenge, and shared creative experience (Hu et al., 2023; Nikolopoulou, 2025; Su et al., 2024). Passive AI instruction, which positions children as recipients of AI-generated content, is theorised to thwart need satisfaction by reducing choice, challenge, and interpersonal interaction (Kanders et al., 2024; Strouse et al., 2018). SDT research consistently shows that autonomous motivation is the proximal process through which environmental conditions translate into engagement quality (Chiu, 2021; Vansteenkiste et al., 2020). We therefore hypothesise autonomous motivation as the mediating mechanism:

**H2a.**  *Autonomous motivation mediates the positive association between AI-enhanced interactive pedagogy and creative learning engagement.*

**H2b.**  *Autonomous motivation mediates the negative association between AI-dependent passive instruction and creative learning engagement.*

### 2.4. Perceived Teacher–Child Interaction Quality as a Buffering Moderator

Perceived teacher–child interaction quality, as conceptualised in CLASS, encompasses emotional support, classroom organisation, and instructional support (Hamre et al., 2013; Pianta & Hofkens, 2023). A substantial evidence base links higher CLASS scores to children’s academic gains, self-regulation and creative expression (Hamre et al., 2013; Pianta & Hofkens, 2023). The present study uses self-reported rather than observationally measured CLASS scores, and this distinction is maintained throughout.

High-quality interaction may provide a relationally grounded source of need satisfaction that compensates for the need-thwarting properties of passive technology use. Warm emotional support may sustain children’s relatedness even during passive screen exposure. Effective instructional scaffolding may restore the competence challenge that passive exposure alone does not provide. And proactive classroom organisation may bound passive AI episodes with active exploration (Hu et al., 2023; Li et al., 2026; Pianta & Hofkens, 2023). This argument is consistent with SDT’s account of contextual need support buffering motivational costs (Ryan & Deci, 2017; Vansteenkiste et al., 2020), CLASS-based evidence on interaction quality compensating for structural disadvantage (Hamre et al., 2013; Pianta & Hofkens, 2023), and evidence that teacher presence moderates GenAI-based learning outcomes (Li et al., 2025).

Complementary evidence from the developmental science of children’s interactions with AI-based technologies further supports the importance of teacher mediation. Research on young children’s (aged 4–6) evaluation of scientific explanations from digital voice assistants, teachers, and peers has shown that children tend to trust AI-generated explanations regardless of explanation quality, whereas they apply quality-based evaluation criteria more consistently to human informants (Haber et al., 2026). This differential trust pattern suggests that young children lack the critical evaluation skills needed to independently assess AI-produced content, making teacher scaffolding and high-quality teacher–child interaction especially important when GenAI tools are used in preschool settings. When teachers maintain warm, responsive, and cognitively stimulating interactions around AI-mediated activities, they can model the evaluative stance that children do not yet spontaneously apply to technological sources, thereby compensating for the motivational risks of passive AI exposure.

Interactive AI pedagogy and high-quality teacher–child interaction likely operate through overlapping need support mechanisms, leading to additive rather than interactive associations (Nikolopoulou, 2025; Su et al., 2024). We therefore propose:

**H3.**  *Perceived teacher–child interaction quality moderates the indirect association between AI-dependent passive instruction and children’s creative learning engagement via autonomous motivation, such that the negative indirect association is attenuated at higher levels of perceived teacher–child interaction quality.*

## 3. Methodology

### 3.1. Participants and Procedure

Following a pilot study (n=86), data were collected between November 2025 and February 2026 across 52 kindergartens in three Chinese provinces (Shandong, Henan, and Sichuan), purposively sampled to represent eastern, central, and western regions. An online survey was distributed to preschool teachers who had used GenAI tools at least once during the preceding semester. Each teacher reported on their own practices and rated a randomly selected focal child. Of 538 returned questionnaires, 52 were excluded, yielding N=486.

The sample composition is summarised in Table 1. The majority were female (95.1%), consistent with the Chinese preschool workforce.

Table 2 reports GenAI use frequency. Most teachers used GenAI tools on a weekly basis, predominantly LLM chatbots, with typical sessions lasting 10–20 min.

The study was approved by the institutional ethics review board. To reduce common method bias, scale order was randomised, and the perceived interaction quality scale was separated from GenAI scales by demographic and filler items (Podsakoff et al., 2003).

### 3.2. Measures

All scales were administered in Mandarin Chinese, with established English instruments translated using forward–backward translation. All items used a five-point Likert scale (1 = strongly disagree/never to 5 = strongly agree/always) unless otherwise indicated.

#### 3.2.1. GenAI-Assisted Teaching Practices

A ten-item scale was developed based on GenAI in early childhood education literature (Kanders et al., 2024; Nikolopoulou, 2025; Su & Yang, 2024) and the active versus passive technology use distinction (Herodotou, 2018; Strouse et al., 2018). An initial 18-item pool was generated from the literature review and teacher interviews, reviewed by a five-member expert panel (content validity index threshold 0.80), and refined to 12 items. Pilot EFA (n=86, oblimin rotation yielded a two-factor solution; two items with cross-loadings above 0.35 were removed, producing the final ten-item scale. The AI-enhanced interactive pedagogy subscale (five items; example: “I use AI tools to co-create stories or art activities with children, encouraging them to contribute their own ideas”) and the AI-dependent passive instruction subscale (five items; example: “I sometimes let AI-generated content replace direct teacher–child interaction during learning activities”) showed satisfactory discriminant validity against a general technology integration scale (r=0.38 and r=0.29, respectively).

#### 3.2.2. Children’s Autonomous Motivation

A ten-item teacher-rated scale adapted from the Self-Regulation Questionnaire (Ryan & Connell, 1989) contextualised for preschool learning (example: “This child participates in learning activities because they find them genuinely interesting and enjoyable”), was used. The unidimensional structure was confirmed in the main sample.

#### 3.2.3. Perceived Teacher–Child Interaction Quality

A twelve-item self-report scale adapted from CLASS (Hamre et al., 2013; Pianta & Hofkens, 2023) spanned emotional support, classroom organisation, and instructional support. Teacher self-report CLASS adaptations have been used in prior large-scale survey research (Hu et al., 2023; Li et al., 2026).

#### 3.2.4. Children’s Creative Learning Engagement

A twelve-item teacher-rated scale integrating engagement dimensions (Fredricks et al., 2004) with creativity indicators (Amabile & Pillemer, 2012) captured sustained attention, novel idea generation, and positive emotional expression (example: “This child generates original ideas during creative activities”).

#### 3.2.5. Control Variables

Teacher gender, teaching experience, educational attainment, kindergarten type, focal child age group, and region were included as control variables.

### 3.3. Data Analysis Strategy

Because 486 teachers were nested within 52 kindergartens, ICCs and design effects were computed (Kline, 2023). The measurement model was evaluated by CFA with robust ML estimation; fit was assessed via CFI, TLI, RMSEA, and SRMR. Composite reliability, AVE, and the Fornell–Larcker criterion assessed reliability and discriminant validity (Fornell & Larcker, 1981). Common method bias was checked via Harman’s single-factor test and a marker variable (Podsakoff et al., 2003). Conditional indirect effects were estimated using bias-corrected bootstrap with 5000 resamples, probed at the mean and ±1 SD of perceived interaction quality (Hayes, 2018). All analyses were conducted in Mplus 8.10 and SPSS 27.0 with PROCESS v4.2.

## 4. Results

### 4.1. Preliminary Analyses

ICCs ranged from 0.04 to 0.07 across model variables. With an average cluster size of c=9.35, design effects ranged from 1.33 to 1.58, below the 2.0 threshold for requiring multilevel modelling (Kline, 2023). Supplementary analysis with TYPE = COMPLEX in Mplus produced unchanged conclusions (coefficient changes below 0.02). Harman’s single-factor test yielded a largest factor accounting for 26.7% of variance (<50% threshold), and the marker variable correlation was 0.05, suggesting common method bias does not dominate the findings (Podsakoff et al., 2003). These safeguards are acknowledged as relatively weak; residual shared-rater concerns are discussed in the Limitations.

Table 3 reports descriptive statistics and correlations. All bivariate correlations were in the expected directions and consistent with prior research (Chiu, 2021; Guay, 2022).

Square roots of AVE are on the diagonal, all exceeding corresponding off-diagonal correlations, supporting discriminant validity (Fornell & Larcker, 1981). Descriptive statistics for perceived teacher–child interaction quality by CLASS domain are presented in Table 4.

The descending pattern of means across domains is consistent with observational CLASS norms, although absolute values are higher, likely reflecting self-report positivity bias.

### 4.2. Measurement Model

The five-factor CFA demonstrated acceptable fit: $\chi^{2}\left(692\right)=1563.84$, $\chi^{2}/df=2.26$, CFI =0.941, TLI =0.936, RMSEA =0.051 (90% CI [0.047, 0.055]), SRMR =0.043. Alternative models (single-factor, three-factor, four-factor) fit substantially worse, supporting construct distinctiveness (Kline, 2023). Reliability and convergent validity indices are in Table 5; all Cronbach’s α (0.86–0.93), CR (0.89–0.94), and AVE (0.58–0.69) exceeded conventional thresholds. Standardised factor loadings are in Table 6.

### 4.3. Structural Path Analysis

**Figure 2:**
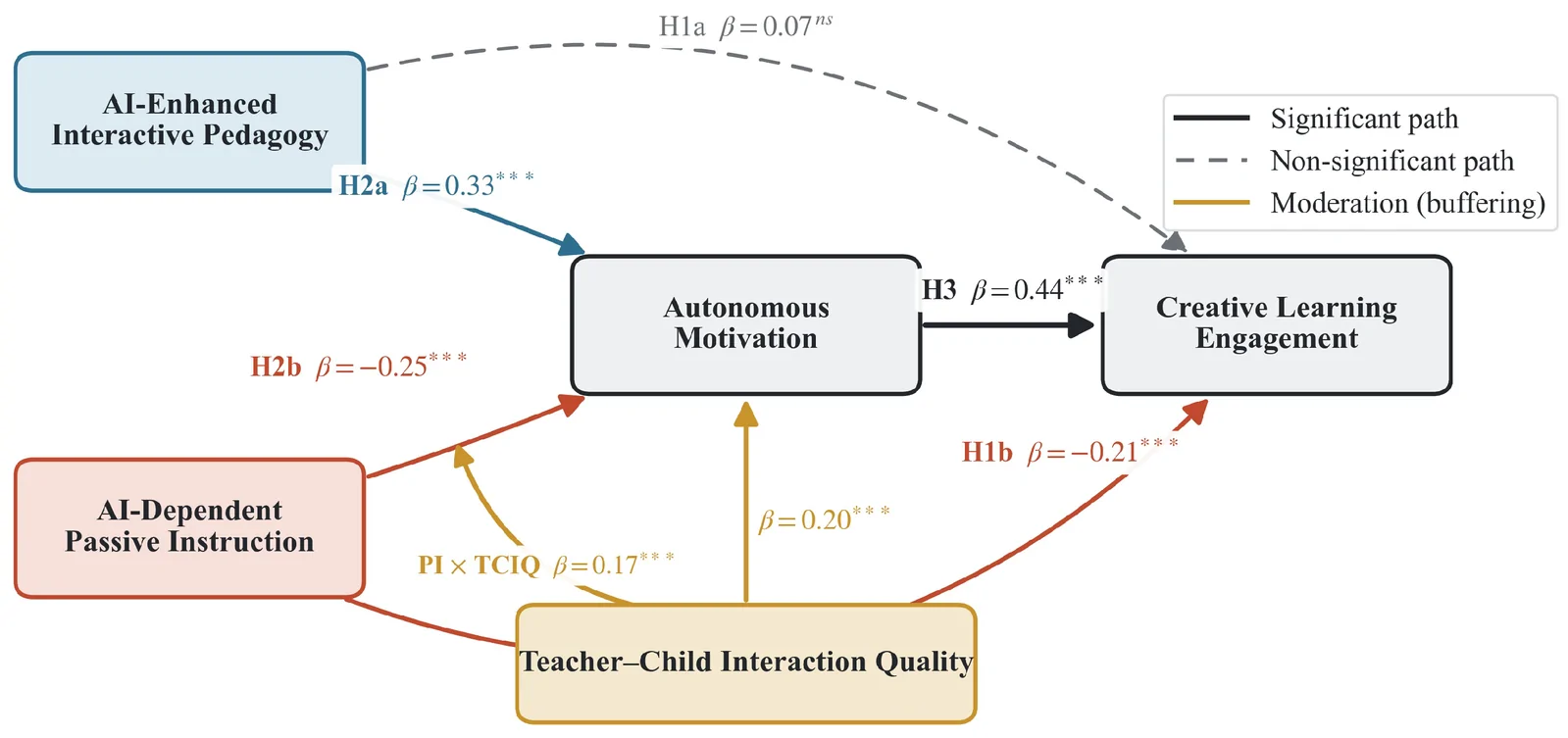
Standardised path coefficients of the moderated mediation model (N=486). *** p<0.001; ns = not significant.

The structural model fit the data well: $\chi^{2}\left(706\right)=1598.58$, $\chi^{2}/df=2.26$, CFI =0.939, TLI =0.935, RMSEA =0.051, SRMR =0.047. Standardised path coefficients are in Table 7 and Figure 2.

Consistent with H1b, passive instruction was negatively associated with creative learning engagement (β=−0.21, p<0.001). The direct path from interactive pedagogy to engagement was not significant (β=0.07, p=0.134), although it operated indirectly via autonomous motivation (Section 4.4). The interaction between passive instruction and perceived interaction quality on autonomous motivation was significant (β=0.17, p<0.001); together with the negative main association (β=−0.25), the positive interaction indicates a buffering pattern. The parallel interaction with interactive pedagogy was not significant (β=0.05, p=0.198).

### 4.4. Mediation Analysis

**Figure 3:**
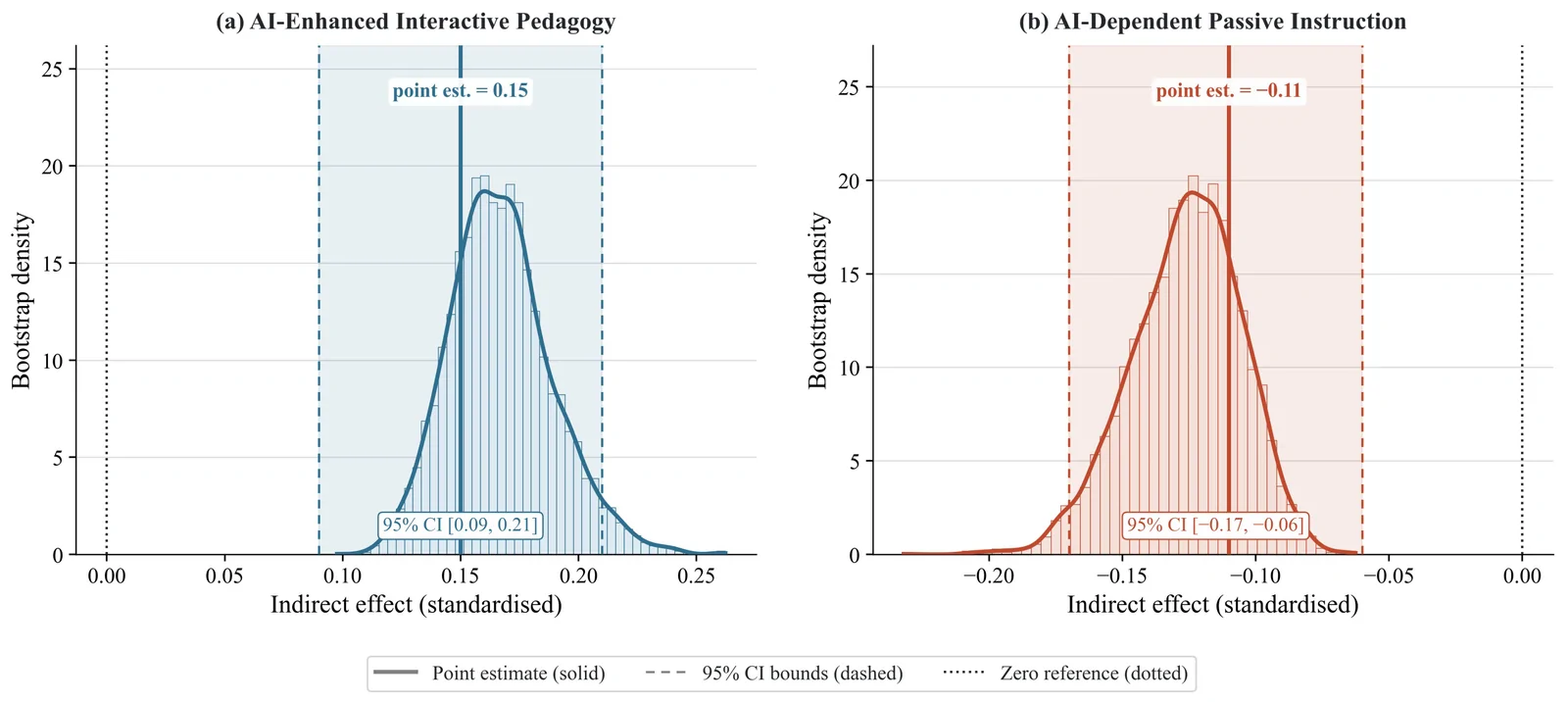
Bootstrap distributions of indirect effects via autonomous motivation. (**a**) AI-Enhanced Interactive Pedagogy (point est. =0.15, 95% CI [0.09,0.21]); (**b**) AI-Dependent Passive Instruction (point est. =−0.11, 95% CI [−0.17,−0.06]). Solid vertical lines indicate point estimates; dashed vertical lines indicate 95% CI bounds; dotted vertical lines indicate the zero reference. Shaded regions represent the 95% CI interval. All estimates are based on N=5000 bootstrap resamples.

Table 8 and Figure 3 present bootstrap results (5000 resamples). Autonomous motivation fully mediated the interactive pedagogy to engagement association (indirect =0.15, 95% CI [0.09, 0.21]) and partially mediated the passive instruction to engagement association (indirect =−0.11, 95% CI [−0.17, −0.06]), supporting H2a and H2b.

### 4.5. Moderated Mediation Analysis

**Figure 4:**
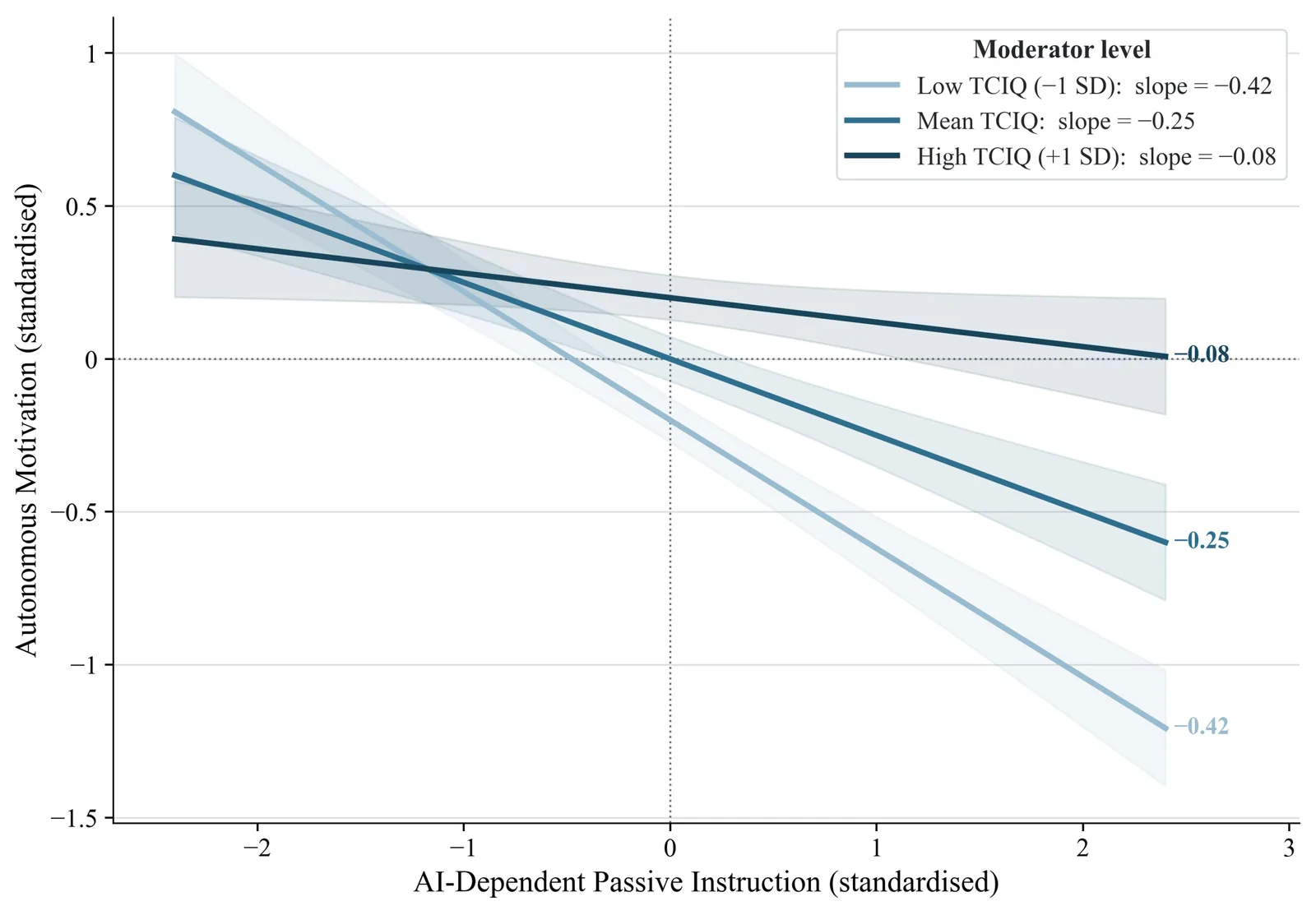
Simple slopes of passive instruction predicting autonomous motivation at ±1 SD of perceived interaction quality.

**Figure 5:**
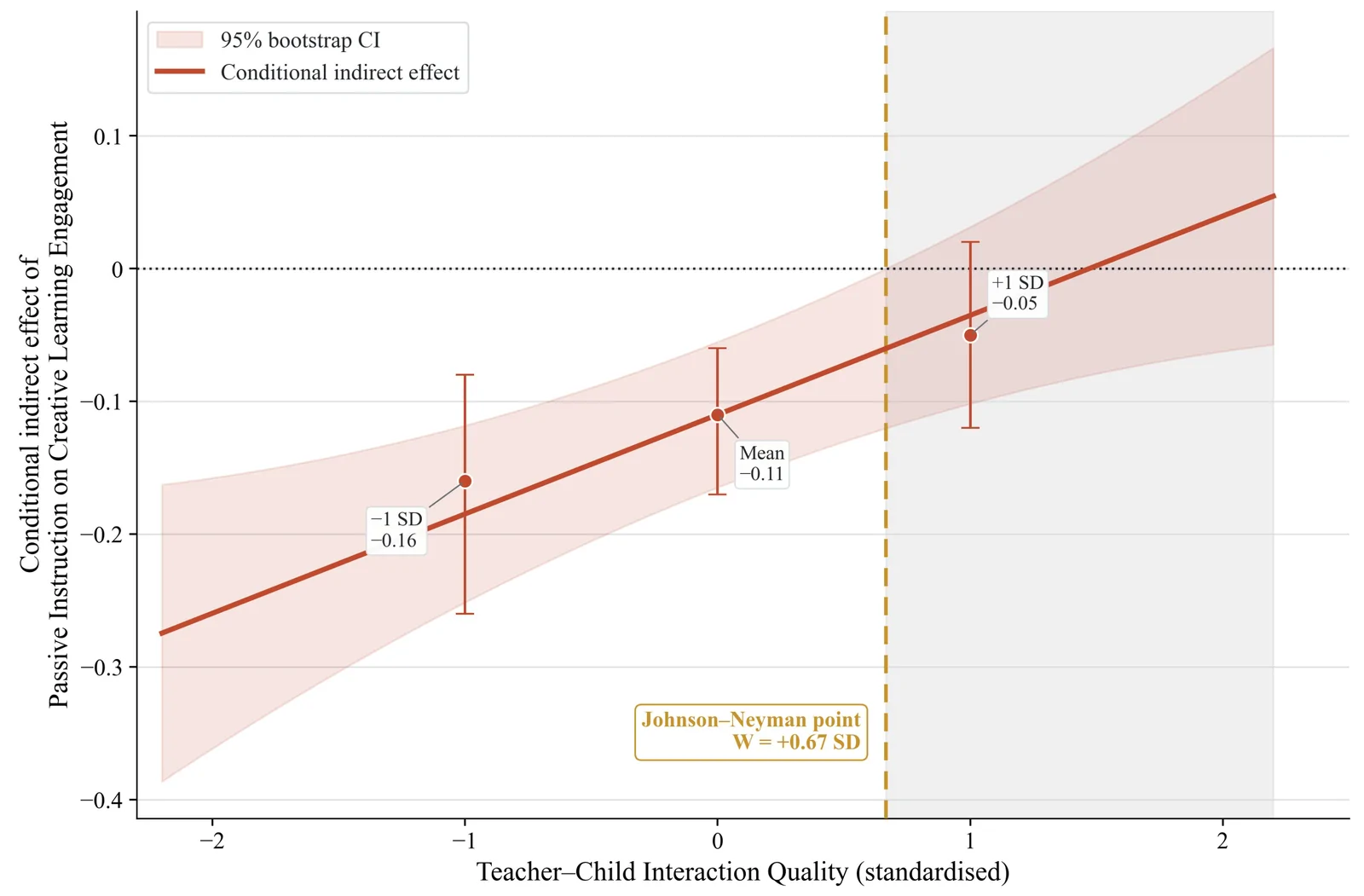
Conditional indirect association of passive instruction with creative learning engagement across perceived interaction quality levels.

The index of moderated mediation was 0.07 (95% bootstrap CI [0.03, 0.12]), supporting H3. Conditional indirect effects are in Table 9; simple slopes are in Figure 4, and the Johnson–Neyman boundary is in Figure 5. At low perceived interaction quality (−1 SD), the indirect association was substantial (−0.16, 95% CI [−0.24, −0.10]); at the mean, it was attenuated (−0.11, 95% CI [−0.17, −0.06]); at high levels (+1 SD), it was no longer significant (−0.05, 95% CI [−0.11, 0.01]). The parallel index for interactive pedagogy was not significant (0.02, 95% CI [−0.01, 0.06]).

The index equals the product of the interaction coefficient and the motivation-to-engagement path: (+0.17)×(0.44)≈0.07.

The Johnson–Neyman significance boundary indicates the level of perceived interaction quality at which the indirect association transitions from significant to non-significant.

### 4.6. Supplementary Analyses

**Figure 6:**
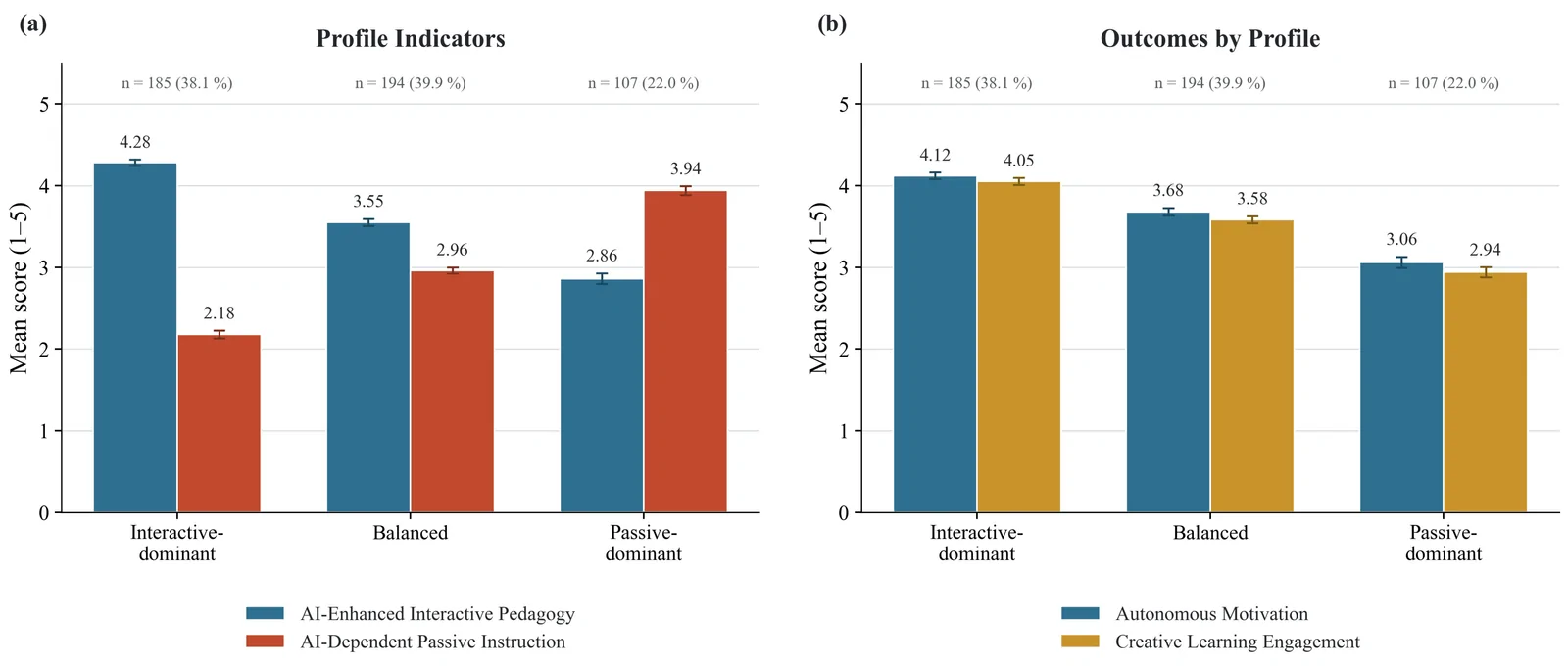
Mean scores across three latent profiles of GenAI teaching practices (N=486). (**a**) Profile-defining indicators: mean scores of AI-Enhanced Interactive Pedagogy and AI-Dependent Passive Instruction across the three profiles; (**b**) developmental outcomes: mean scores of Autonomous Motivation and Creative Learning Engagement across the three profiles. Error bars represent ±1 SE.

Robustness was supported across several checks. The model was re-estimated dropping each control variable in turn, with unchanged conclusions. Subgroup analyses by kindergarten type, child age, and region yielded interaction coefficients ranging from 0.12 to 0.21 (all significant). An alternative specification treating perceived interaction quality as a mediator yielded inferior fit ($\Delta \chi^{2}=24.8$, Δdf=3, p<0.001). Latent profile analysis identified three GenAI teaching profiles (interactive-dominant, balanced, passive-dominant; Figure 6), with motivation and engagement comparisons paralleling the variable-centred findings.

## 5. Discussion

### 5.1. Asymmetric Associations of GenAI Teaching Modes

AI-enhanced interactive pedagogy and AI-dependent passive instruction operated asymmetrically: passive instruction was directly associated with lower engagement (β=−0.21), whereas interactive pedagogy’s association was entirely transmitted through autonomous motivation. This asymmetry echoes developmental evidence that active and passive technology use differ qualitatively (Herodotou, 2018; Strouse et al., 2018) and extends this distinction to GenAI-assisted preschool teaching. The direct negative association of passive instruction suggests additional disengagement mechanisms beyond the motivational route, plausibly including reduced hands-on exploration and displacement of peer interaction (Kanders et al., 2024; Nikolopoulou, 2025). These findings extend evidence from Chinese higher education, where GenAI use is associated with increased engagement but may simultaneously reduce active learning activities (Guo et al., 2025; Hon, 2026), to the preschool context, demonstrating that the mode of GenAI use matters more than its frequency. Promoting interactive GenAI use alone is insufficient; explicit attention to reducing passive, substitutive AI use is also required. The cross-sectional design does not permit causal inference; children who are already motivated may elicit more interactive AI teaching, while disengaged children may prompt teachers to resort to passive AI content.

### 5.2. Autonomous Motivation as a Differentiating Mechanism

Consistent with H2a and H2b, autonomous motivation mediated both GenAI teaching pathways with theoretically important differences. Full mediation of interactive pedagogy suggests that the motivational system is the dominant route for interactive AI use, whereas partial mediation of passive instruction indicates additional non-motivational routes. This aligns with SDT’s proposition that autonomous motivation is the primary process through which need-supportive environments translate into high-quality engagement (Ryan & Deci, 2017; Vansteenkiste et al., 2020), and is consistent with evidence that intrinsic motivation mediates the relationship between AI-supported classroom innovation and student creativity (Jiang et al., 2025). These results must be interpreted with caution given the single-informant design: the same teacher rated the predictor, mediator, and outcome, so the observed mediation may partly reflect cognitive consistency rather than actual psychological processes within children. Multi-informant designs are needed to disentangle substantive mediation from shared-method artefact. Interventions enriching the motivational environment (e.g., designing GenAI activities with choice and collaborative interaction) will capitalise on the interactive pedagogy pathway but only partially shield against passive AI exposure. Complementary interventions targeting non-motivational routes, such as counterbalancing AI content with physical play and structuring digital-to-hands-on transitions, are also needed.

### 5.3. Perceived Teacher–Child Interaction Quality as a Contextual Buffer

Consistent with H3, the negative indirect association of passive AI instruction with creative learning engagement via autonomous motivation was substantially attenuated at high perceived interaction quality and non-significant at the upper level. The asymmetry of the moderation supports the need-compensation mechanism: high-quality interaction provides a relationally grounded source of need satisfaction that specifically counteracts the need-thwarting properties of passive AI exposure. This pattern is consistent with SDT’s account of contextual need support (Ryan & Deci, 2017; Vansteenkiste et al., 2020), CLASS-based evidence on interaction quality compensating for structural disadvantage (Hamre et al., 2013; Pianta & Hofkens, 2023), and evidence that teacher presence moderates GenAI learning outcomes (Li et al., 2025).

The present buffering pattern also resonates with developmental evidence on young children’s interactions with AI-based information sources. Research has demonstrated that preschool-aged children (4–6 years) tend to trust scientific explanations from digital voice assistants regardless of explanation quality, in contrast to their more evaluative stance toward human informants (Haber et al., 2026). This asymmetric trust pattern underscores why passive AI exposure may be particularly detrimental in the absence of teacher mediation: young children are unlikely to critically filter AI-generated content on their own. Teachers who maintain high-quality interactions can scaffold the evaluative skills that children do not yet spontaneously apply to technological sources, providing the relational and cognitive infrastructure that compensates for the need-thwarting properties of passive AI instruction.

A critical qualification concerns the self-report operationalisation: the buffering pattern may partly reflect a cognitive-perceptual process in which teachers who view themselves as providing high-quality interactions also perceive children as less negatively affected. Future studies should replicate the pattern using observational CLASS ratings. Theoretically, this positions perceived interaction quality as a specific buffer against maladaptive technology-related practices, refining the SDT account of contextual need support in technology-rich environments. Practically, investing in CLASS-based professional development is a more promising avenue than restricting technology access, as it may preserve the benefits of interactive AI use while attenuating the costs of passive use.

### 5.4. Practical Implications

Teacher training should be bidirectional: structured professional development should help teachers maximise interactive, co-creative AI use (Nikolopoulou, 2025; Su & Yang, 2024), while reflective practice should help them identify and reduce passive AI episodes. The motivational pathway should be a central design principle: AI activities should support children’s autonomy, competence, and relatedness rather than deliver content efficiently. CLASS-based professional development should be scaled alongside technology adoption, with the quality of teacher–child interaction surrounding technology use as the primary improvement target. The findings should not place the entire burden on individual teachers; technology developers and policymakers should invest in GenAI tools designed for early childhood contexts that promote teacher–child co-engagement and embed safeguards against passive screen exposure. Policy frameworks should include design standards for educational AI tools alongside teacher professional development.

### 5.5. Developmental Appropriateness Considerations

The sample included children aged three to six, a span encompassing substantial cognitive and socioemotional development. Younger children may be more vulnerable to passive AI exposure due to fewer self-regulatory resources, while older preschoolers may be better equipped to benefit from interactive AI activities. Although subgroup analyses showed a consistent buffering pattern, the study was not powered to test age-moderated effects, and future research should explicitly examine developmental level as a moderator.

## 6. Conclusions, Limitations, and Future Directions

This study tested a moderated mediation model linking two dimensions of GenAI-assisted teaching to children’s creative learning engagement through autonomous motivation, with perceived teacher–child interaction quality as a boundary condition. GenAI teaching operates asymmetrically: passive instruction shows a direct negative association with engagement, whereas interactive pedagogy operates indirectly through motivation. Autonomous motivation differentially mediates the two pathways, and perceived interaction quality attenuates the passive instruction pathway to non-significance at high levels. These findings extend SDT to the GenAI era and offer preliminary guidance for teacher-scaffolded GenAI integration in preschool education. All findings are based on teacher self-report and should be interpreted as reflecting teachers’ perceptions rather than causal relationships.

The most important limitation is the single-informant design, which creates a risk that observed associations are inflated by common-rater variance (Podsakoff et al., 2003). Future research should prioritise multi-informant designs combining teacher ratings with independent classroom observations, parent reports, and performance-based creativity assessments. The cross-sectional design precludes causal inference; multi-wave longitudinal or quasi-experimental designs tracking GenAI practices, motivation, and engagement over time would strengthen the evidence base. The self-report CLASS adaptation may capture a construct distinct from observational CLASS scores; future studies should employ observational CLASS assessments. The GenAI teaching practices scale requires external validation across diverse contexts. Future studies should consider additional mediators (e.g., self-regulation, creative self-efficacy) and moderators (e.g., parental technology attitudes, children’s temperament, age within the preschool range), and disaggregate autonomous motivation into its constituent subtypes. The Chinese context, with its centralised policy framework and substantial technology infrastructure investment, may limit generalisability; cross-national comparative research is needed. The design features of specific GenAI tools were not examined and warrant future investigation. Future studies with larger kindergarten samples should employ multilevel structural equation modelling to partition teacher- and school-level variance.

Despite these limitations, the study provides initial evidence that the mode of GenAI use matters more than its presence, that the motivational pathway is primary for interactive AI use, and that perceived teacher–child interaction quality is associated with the attenuation of passive AI instruction’s negative correlates. These findings reposition the teacher as the decisive agent in GenAI-assisted preschool education. Replication with multi-informant, longitudinal, and observational designs is essential before these associations can inform causal policy guidance.

## Figures and Tables

**Table 1:** Sample composition (N=486).

Characteristic	*n*	%
Gender, Female	462	95.1
Gender, Male	24	4.9
Teaching experience, <3 years	137	28.2
Teaching experience, 3–5 years	160	32.9
Teaching experience, 6–10 years	117	24.1
Teaching experience, >10 years	72	14.8
Education, Associate degree	170	35.0
Education, Bachelor’s degree	257	52.9
Education, Master’s degree or above	59	12.1
Kindergarten type, Public	285	58.6
Kindergarten type, Private	201	41.4
Focal child age, 3–4 years	163	33.5
Focal child age, 4–5 years	170	35.0
Focal child age, 5–6 years	153	31.5
Region, Eastern	166	34.2
Region, Central	161	33.1
Region, Western	159	32.7

**Table 2:** Frequency and type of GenAI use among participating teachers (N=486).

Characteristic	*n*	%
Frequency of GenAI use		
Daily	71	14.6
Several times per week	194	39.9
About once per week	138	28.4
Less than once per week	83	17.1
Primary GenAI tool type		
LLM chatbot (e.g., ChatGPT (GPT-3.5), Doubao (v1.5), Wenxin Yiyan (ERNIE Bot 4.0))	263	54.1
AI image/art generator	124	25.5
AI story/narrative generator	67	13.8
Other (AI music, AI coding tools, etc.)	32	6.6
Typical session duration		
Less than 10 min	89	18.3
10–20 min	227	46.7
21–30 min	131	27.0
More than 30 min	39	8.0

**Table 3:** Descriptive statistics, correlations, and discriminant validity.

Variable	*M*	*SD*	1	2	3	4	5
1. AI-enhanced interactive pedagogy	3.58	0.79	0.78				
2. AI-dependent passive instruction	2.94	0.88	−0.16 ***	0.76			
3. Autonomous motivation	3.71	0.84	0.43 ***	−0.34 ***	0.83		
4. Perceived teacher–child interaction quality	3.82	0.81	0.32 ***	−0.14 **	0.36 ***	0.80	
5. Creative learning engagement	3.65	0.86	0.31 ***	−0.38 ***	0.53 ***	0.27 ***	0.81

Note. N=486. ** *p* < 0.01; *** *p* < 0.001.

**Table 4:** Descriptive statistics for perceived teacher–child interaction quality by CLASS domain.

CLASS Domain	Items	*M*	*SD*	Skewness	Kurtosis	α
Emotional support	4	4.01	0.76	−0.58	0.21	0.87
Classroom organisation	4	3.78	0.83	−0.41	−0.12	0.85
Instructional support	4	3.67	0.85	−0.34	−0.19	0.84
Overall perceived interaction quality	12	3.82	0.81	−0.44	−0.03	0.89

**Table 5:** Confirmatory factor analysis: reliability and convergent validity.

Construct	Cronbach’s α	CR	AVE
AI-enhanced interactive pedagogy	0.88	0.90	0.61
AI-dependent passive instruction	0.86	0.89	0.58
Autonomous motivation	0.91	0.93	0.69
Perceived teacher–child interaction quality	0.89	0.91	0.63
Creative learning engagement	0.93	0.94	0.66

**Table 6:** Standardised factor loadings from confirmatory factor analysis.

Item	Loading
AI-enhanced interactive pedagogy	
IP1: Co-create stories or art with children using AI tools	0.81
IP2: Use AI to generate prompts for children’s creative exploration	0.79
IP3: Integrate AI-generated materials into dialogic group activities	0.77
IP4: Use AI tools to scaffold children’s problem-solving during play	0.76
IP5: Facilitate child–AI–teacher collaborative activities	0.78
AI-dependent passive instruction	
PI1: Let AI-generated content replace direct teacher–child interaction	0.74
PI2: Allow children to watch AI-generated content without guided discussion	0.76
PI3: Use AI to deliver lessons while reducing own instructional role	0.78
PI4: Rely on AI outputs without adapting them to children’s responses	0.75
PI5: Substitute hands-on activities with AI-generated exercises	0.77
Autonomous Motivation	
AM1–AM10 (range)	0.76–0.87
Perceived Teacher–Child Interaction Quality	
Emotional support items (ES1–ES4)	0.74–0.84
Classroom organisation items (CO1–CO4)	0.72–0.82
Instructional support items (IS1–IS4)	0.73–0.83
Creative Learning Engagement	
CLE1–CLE12 (range)	0.72–0.86

Note. N=486. All p<0.001.

**Table 7:** Standardised path coefficients in the structural model.

Path	β	SE	95% CI	*p*
Interactive pedagogy → Creative learning engagement	0.07	0.05	[−0.02,0.16]	0.134
Passive instruction → Creative learning engagement	−0.21	0.04	[−0.29,−0.13]	<0.001
Interactive pedagogy → Autonomous motivation	0.33	0.04	[0.25,0.41]	<0.001
Passive instruction → Autonomous motivation	−0.25	0.04	[−0.33,−0.17]	<0.001
Autonomous motivation → Creative learning engagement	0.44	0.04	[0.36,0.52]	<0.001
Perceived interaction quality → Autonomous motivation	0.20	0.04	[0.12,0.28]	<0.001
Passive instruction × Perceived interaction quality → AM	0.17	0.04	[0.09,0.25]	<0.001
Interactive pedagogy × Perceived interaction quality → AM	0.05	0.04	[−0.03,0.13]	0.198

Note. N=486. Control variables included but omitted. AM = autonomous motivation.

**Table 8:** Mediation analysis: indirect, direct, and total associations.

Path	Effect	SE	95% Bootstrap CI	Proportion Mediated
Interactive pedagogy → AM → Creative learning engagement				
Indirect via motivation	0.15	0.03	[0.09,0.21]	68.2%
Direct	0.07	0.05	[−0.02,0.16]	n.a.
Total	0.22	0.05	[0.13,0.31]	n.a.
Passive instruction → AM → Creative learning engagement				
Indirect via motivation	−0.11	0.03	[−0.17,−0.06]	34.4%
Direct	−0.21	0.04	[−0.29,−0.13]	n.a.
Total	−0.32	0.05	[−0.41,−0.23]	n.a.

Note. N=486. Bootstrap 5000 resamples. AM = autonomous motivation.

**Table 9:** Conditional indirect effects of passive instruction on creative learning engagement via autonomous motivation.

Perceived Interaction Quality Level	Indirect Effect	Boot SE	95% Bootstrap CI	Significant?
Low (−1 SD)	−0.16	0.04	[−0.24,−0.10]	Yes
Mean	−0.11	0.03	[−0.17,−0.06]	Yes
High (+1 SD)	−0.05	0.03	[−0.11,0.01]	No
Index of moderated mediation	0.07	0.02	[0.03,0.12]	Yes

Note. N=486. Bootstrap 5000 resamples.

## Data Availability

The data presented in this study are available on reasonable request from the corresponding author. The data are not publicly available due to privacy and ethical restrictions.

## References

[B1-behavsci-16-01293] Alfarwan A. (2025). Generative AI use in K-12 education: A systematic review. Frontiers in Education.

[B2-behavsci-16-01293] Amabile T. M., Pillemer J. (2012). Perspectives on the social psychology of creativity. The Journal of Creative Behavior.

[B3-behavsci-16-01293] Berson I. R., Berson M. J. (2024). Fragments of the past: The intersection of AI, historical imagery, and early childhood creativity. Future in Educational Research.

[B4-behavsci-16-01293] Chen J. (2025). Enjoyment of AI-generated stories blending art and science: Impact on preschoolers’ pro-environmental attitudes. Frontiers in Psychology.

[B5-behavsci-16-01293] Chiu T. K. F. (2021). Digital support for student engagement in blended learning based on self-determination theory. Computers in Human Behavior.

[B6-behavsci-16-01293] Chiu T. K. F. (2024). The impact of generative AI (GenAI) on practices, policies and research direction in education: A case of ChatGPT and Midjourney. Interactive Learning Environments.

[B7-behavsci-16-01293] Fornell C., Larcker D. F. (1981). Evaluating structural equation models with unobservable variables and measurement error. Journal of Marketing Research.

[B8-behavsci-16-01293] Fredricks J. A., Blumenfeld P. C., Paris A. H. (2004). School engagement: Potential of the concept, state of the evidence. Review of Educational Research.

[B9-behavsci-16-01293] Guay F. (2022). Applying self-determination theory to education: Regulations types, psychological needs, and autonomy supporting behaviors. Canadian Journal of School Psychology.

[B10-behavsci-16-01293] Guo F., Zhang L., Shi T., Coates H. (2025). Whether and when could generative AI improve college student learning engagement?. Behavioral Sciences.

[B11-behavsci-16-01293] Haber A. S., Kumar S. C., Swenson M., Bode K., Ruel E. (2026). How do children evaluate scientific explanations provided by digital voice assistants, teachers, and peers?. Behavioral Sciences.

[B12-behavsci-16-01293] Hamre B. K., Pianta R. C., Downer J. T., DeCoster J., Mashburn A. J., Jones S. M., Brown J. L., Cappella E., Atkins M., Rivers S. E., Brackett M. A., Hamagami A. (2013). Teaching through interactions: Testing a developmental framework of teacher effectiveness in over 4000 classrooms. The Elementary School Journal.

[B13-behavsci-16-01293] Hayes A. F. (2018). Introduction to mediation, moderation, and conditional process analysis: A regression-based approach.

[B14-behavsci-16-01293] Herodotou C. (2018). Young children and tablets: A systematic review of effects on learning and development. Journal of Computer Assisted Learning.

[B15-behavsci-16-01293] Hon K. K. L. (2026). Generative AI in higher education: A systematic review of its effects on learning outcomes and academic performance. Journal of Educational Technology Systems.

[B16-behavsci-16-01293] Hu X., Chiu M. M., Yelland N., Liang Y. (2023). Scaffolding young children’s computational thinking with teacher talk in a technology-mediated classroom. Early Childhood Research Quarterly.

[B17-behavsci-16-01293] Jiang C., Han M., Mao Z., Yu J., Jiang F., Liang Y. (2025). Classroom innovation atmosphere and student creativity: The roles of AI technology application, intrinsic motivation, and self-efficacy. Frontiers in Psychology.

[B18-behavsci-16-01293] Kanders K., Stupple-Harris L., Smith L., Gibson J. L. (2024). Perspectives on the impact of generative AI on early-childhood development and education. Infant and Child Development.

[B19-behavsci-16-01293] Kline R. B. (2023). Principles and practice of structural equation modeling.

[B20-behavsci-16-01293] Li H., He H., Luo W., Li H. (2026). Early childhood digital pedagogy: A scoping review of its practices, profiles, and predictors. Early Childhood Education Journal.

[B21-behavsci-16-01293] Li Y., Wu Y., Chiu T. K. F. (2025). How teacher presence affects student engagement with a generative artificial intelligence chatbot in learning designed with first principles of instruction. Journal of Research on Technology in Education.

[B22-behavsci-16-01293] Liang J., Wang L., Luo J., Yan Y., Fan C. (2023). The relationship between student interaction with generative artificial intelligence and learning achievement: Serial mediating roles of self-efficacy and cognitive engagement. Frontiers in Psychology.

[B23-behavsci-16-01293] Ljungcrantz L. (2026). The interaction of AI and early childhood education: A state-of-the-art review 2020–2024. Early Childhood Education Journal.

[B24-behavsci-16-01293] Nikolopoulou K. (2025). Child-centered integration of generative AI in early learning: Balancing promises and challenges. AI, Brain and Child.

[B25-behavsci-16-01293] Pianta R. C., Hofkens T. (2023). Defining early education quality using CLASS-observed teacher-student interaction. Frontiers in Psychology.

[B26-behavsci-16-01293] Podsakoff P. M., MacKenzie S. B., Lee J. Y., Podsakoff N. P. (2003). Common method biases in behavioral research: A critical review of the literature and recommended remedies. Journal of Applied Psychology.

[B27-behavsci-16-01293] Ryan R. M., Connell J. P. (1989). Perceived locus of causality and internalization: Examining reasons for acting in two domains. Journal of Personality and Social Psychology.

[B28-behavsci-16-01293] Ryan R. M., Deci E. L. (2017). Self-determination theory: Basic psychological needs in motivation, development, and wellness.

[B29-behavsci-16-01293] Strouse G. A., Troseth G. L., O’Doherty K. D., Saylor M. M. (2018). Co-viewing supports toddlers’ word learning from contingent and noncontingent video. Journal of Experimental Child Psychology.

[B30-behavsci-16-01293] Su J., Yang W. (2022). Artificial intelligence in early childhood education: A scoping review. Computers and Education: Artificial Intelligence.

[B31-behavsci-16-01293] Su J., Yang W. (2024). Powerful or mediocre? Kindergarten teachers’ perspectives on using ChatGPT in early childhood education. Interactive Learning Environments.

[B32-behavsci-16-01293] Su J., Yang W., Yim I. H. Y., Li H., Hu X. (2024). Early artificial intelligence education: Effects of cooperative play and direct instruction on kindergarteners’ computational thinking, sequencing, self-regulation and theory of mind skills. Journal of Computer Assisted Learning.

[B33-behavsci-16-01293] Vansteenkiste M., Ryan R. M., Soenens B. (2020). Basic psychological need theory: Advancements, critical themes, and future directions. Motivation and Emotion.

